# Ano1/TMEM16A Overexpression Is Associated with Good Prognosis in PR-Positive or HER2-Negative Breast Cancer Patients following Tamoxifen Treatment

**DOI:** 10.1371/journal.pone.0126128

**Published:** 2015-05-11

**Authors:** Huizhe Wu, Shu Guan, Mingli Sun, Zhaojin Yu, Lin Zhao, Miao He, Haishan Zhao, Weifan Yao, Enhua Wang, Feng Jin, Qinghuan Xiao, Minjie Wei

**Affiliations:** 1 Department of Pharmacology, School of Pharmacy, China Medical University, Shenyang, Liaoning, P. R. China; 2 Department of Breast Surgery, First Hospital of China Medical University, Shenyang, Liaoning, P. R. China; 3 Institute of Pathology and Pathophysiology, First Hospital and College of Basic Medical Sciences of China Medical University, Shenyang, Liaoning, P. R. China; 4 Department of Ion Channel Pharmacology, School of Pharmacy, China Medical University, Shenyang, Liaoning, P. R. China; INRS, CANADA

## Abstract

The calcium-activated chloride channel Ano1 (TMEM16A) is overexpressed in many tumors. Although Ano1 overexpression is found in breast cancer due to 11q13 amplification, it remains unclear whether signaling pathways are involved in Ano1 overexpression during breast cancer tumorigenesis *in vivo*. Estrogen receptor (ER), progesterone receptor (PR), and human epidermal growth factor receptor 2 (HER2) have been known to contribute to breast cancer progression. It is unclear whether Ano1 is associated with clinical outcomes in breast cancer patients with different ER, PR and HER2 status. In the present study, we investigated the Ano1 expression in 431 patients with invasive ductal breast carcinoma and 46 patients with fibroadenoma, using immunohistochemistry, and analyzed the association between Ano1 expression and clinical characteristics and outcomes of breast cancer patients with different ER, PR, and HER2 status. Ano1 was overexpressed in breast cancer compared with fibroadenoma. Ano1 was significantly more associated with breast cancer with the lower clinical stage (stage I or II), or triple-negative status. Mostly importantly, Ano1 overexpression was associated with good prognosis in patients with the PR-positive or HER2-negative status, and in patients following tamoxifen treatment. Multivariate Cox regression analysis showed that Ano1 overexpression was a prognostic factor for longer overall survival in PR-positive or HER2-negative patients, and a predictive factor for longer overall survival in patients following tamoxifen treatment. Our findings suggest that Ano1 may be a potential marker for good prognosis in PR-positive or HER2-negative patients following tamoxifen treatment. The PR and HER2 status defines a subtype of breast cancer in which Ano1 overexpression is associated with good prognosis following tamoxifen treatment.

## Introduction

Anoctamin 1 (Ano1, TMEM16A) is one of 10 members in the Ano family that are involved in a variety of functions including ion transport and phospholipid scrambling [1]. Ano1 and Ano2 are generally believed to be calcium-activated chloride channels (CaCCs), though it remains unclear whether other Ano family members are anion channels [1,2,3,4]. Ano1 is known to be regulated by Ca^2+^ and calmodulin [5,6,7,8,9], and plays important physiological functions including epithelial secretion, neuronal and cardiac excitation, smooth muscle contraction, olfactory and sensory transduction, and pain [1,3,4,10]. Recently, attention has been focused on the role of Ano1 in cancers [11,12,13,14,15].

Before it was identified as a CaCC in 2008 [6,16,17], Ano1 (also known as ORAOV2, DOG1, TAOS2, and FLJ10261) is found to be overexpressed in many cancers such as esophageal squamous cell cancer, gastrointestinal stromal tumor, and head and neck squamous cell carcinoma [18,19,20,21]. Recently, several studies have found overexpression of Ano1 in other tumors including breast cancer [15,22], prostate cancer [13], and chondroblastoma [23]. Ano1 overexpression has been found to be due to amplification of the corresponding chromosomal region in 11q13 [11,15,24,25], and correlates with poor prognosis in patients with breast cancer and head and neck squamous cell carcinoma [15,25].

It remains largely unclear how Ano1 overexpression contributes to tumor malignancy. There are conflicting results regarding the role of Ano1 in cell proliferation in different cells. For example, several studies have shown that Ano1 overexpression promotes cell proliferation in many tumors including head and neck squamous cell carcinoma, breast cancer, and prostate cancer [12,13,15,24]. However, the proliferation-promoting effect of Ano1 is not observed in HEK-293 cells overexpressing various Ano1 isoforms that are identified in breast cancer cells [22]. In addition, Ano1 overexpression has been found to inhibit angiotensin II-induced proliferation in basilar smooth muscle cells [26]. Furthermore, Ano1 has been found to regulate different signaling pathways in different cancer cells. For example, in breast cancer cell lines, Ano1 promotes breast cancer progression by activating the epidermal growth factor receptor (EGFR) and calmodulin-dependent protein kinase (CAMK) signaling pathways [15]. In head and neck squamous cell carcinoma, Ano1 activates the ERK1/2 and increases the levels of cyclin D1 [12].

Recently, Britschgi et al. have found that the Ano1 gene is amplified in breast cancer, and amplification of the Ano1gene is associated with poor prognosis of breast cancer patients [15]. It is unknown whether signaling pathways or transcription factors are involved in Ano1 overexpression in breast cancer tumorigenesis *in vivo*. It is well known that estrogen receptor (ER), progesterone receptor (PR), and human epidermal growth factor receptor 2 (HER2) contribute to breast cancer progression, and ER- and PR-positive breast cancer patients usually receive endocrine treatment [27]. To date, it remains unclear whether Ano1 is associated with the ER, PR, and HER2 status and clinical outcomes in breast cancer patients receiving endocrine treatment. In this study, we investigated the expression of Ano1 in 431 breast cancer patients with invasive ductal carcinoma (IDC). We found that Ano1 overexpression was associated with good prognosis in PR-positive, or HER2-negative patients following tamoxifen treatment. Our study suggests that Ano1 may be a potential marker for good prognosis in breast cancer patients with the PR-positive, or HER2-negative status.

## Materials and Methods

### Ethics statement

The Medical Ethics Committee of China Medical University approved this study. Due to the retrospective nature of the study, the Ethics Committee waived the need of written informed consent by the patients. All the samples were anonymous.

### Patients and tissue samples

Human breast tissue samples were obtained from 431 female patients with IDC and 46 female patients with fibroadenoma, who underwent surgery at the Department of Surgical Oncology and the Department of General Surgery, the First Hospital of China Medical University between January 2008 and December 2009. The average age of the patients with breast cancer was 51 years (range, 20–82 years). The inclusion criteria for breast cancer patients were: 1) IDC; 2) availability of complete clinical data and follow-up status; 3) breast cancer samples were collected for analysis; 4) no previous diagnosis and treatment of breast cancer; and 5) patients who underwent postoperative chemotherapy and/or endocrine therapy. The exclusion criteria were: 1) incomplete clinical data; 2) ER, PR, and HER2 status were not tested, not recorded, or unknown; 3) patients who underwent radiation therapy only without chemotherapy or endocrine therapy; 4) patients who underwent adjuvant trastuzumab treatment; and 5) severe cardiovascular, pulmonary, renal, hepatic, and gastrointestinal diseases. The stage of the cancer (n = 431) was evaluated according to the TNM staging system as follows: stage I (n = 89), stage II (n = 217), and stage IIIA-IIIC (n = 125). The samples were dichotomized into stage I~II (n = 306) and stage IIIA-IIIC (n = 125). The histological grading of the cancer was performed according to the World Health Organization grading system as follows: Grade 1 (n = 90), Grade 2 (n = 397), and Grade 3 (n = 43). Clinicopathological data including patient age, menopausal status, family history of breast cancer, tumor size, lymph node metastasis, ER, PR, and HER2 status, and chemotherapeutic regimes were retrospectively retrieved from medical records. Forty-six breast fibroadenoma samples were used as controls. The average age of the patients with fibroadenoma was 51 years (range, 35–72 years). The diagnosis of breast cancer and fibroadenoma was confirmed by pathological staining. All patients did not undergo radiation therapy, chemotherapy, and hormonal therapy before surgery.

### Breast cancer subtype definition

ER, PR, and HER2 status were classified as positive and negative based on the immunohistochemistry (IHC) results in the medical records. ER-positive and PR-positive status were defined by >1% nuclear staining [28]. HER2 status was recorded on the basis of IHC score as follows: 0, 1+ for negative, 2+ for borderline, and 3+ for positive. We defined HER2-negative and HER2-postive status by 0–1+ staining and 2+-3+ staining, respectively.

### Tissue microarray (TMA) and Immunohistochemistry

Paraffin blocks (donor blocks) containing representative breast cancer samples or fibroadenoma samples were selected by reviewing all of the hematoxylin and eosin-stained slides. Tissue cores with a diameter of 1.5 mm were extracted from each donor block, and precisely arrayed into a new paraffin recipient block with a maximum of 200 cores, using the Organization Microarrayer (Pathology Devices, USA). Sections (4 μm thick) were obtained from formalin-fixed and paraffin-embedded TMA blocks, mounted on poly-L-lysine-coated glass slides, and used for immunohistochemistry.

Sections were deparaffinized with xylene, rehydrated in a graded alcohol series, and washed in distilled water. Sections were then heated in 10 mM sodium citrate buffer (pH 6.0) for 10 min in a microwave oven to retrieve antigen. Endogenous peroxidase activity was blocked by 3% H_2_O_2_ at 37°C for 20 min. Sections were incubated in 10% normal goat serum at 37°C for 30 min to block nonspecific protein binding sites. Sections were then incubated with primary antibodies against Ano1 (Abcam Biotechnology, USA) overnight at 4°C, followed by incubation with biotinylated secondary antibodies for 30 min at 37°C. The slides were then incubated with streptavidin horse-radish peroxidase for additional 30 min (LSAB kit; Dako, Glostrup, Denmark), washed in PBS, and stained with DAB (3, 3-diaminobenzidine). Sections were counterstained with hematoxylin, dehydrated, and mounted. Sections in which primary antibodies were replaced with normal rabbit IgG were used as negative control.

### Evaluation of immunohistochemistry

The immunostaining was examined under a light microscope by two pathologists blinded to the experimental conditions. The intensity of immunoreactivity was scored as follows: 0 for no staining, 1 for weak staining, 2 for moderate staining, and 3 for strong staining. The proportion of tumor cells was calculated as the percentage of Ano1-immunopositive cells over the total tumor cells. Five sections were selected from each sample. For each section, five fields were randomly selected. Scores was assigned by using 5% increments (0%, 5%, 10%, ….100%) as previously reported [29,30]. The average score for each sample was used to assess cutoff scores for Ano1 overexpression, using receiver operating characteristic (ROC) curves. To generate ROC curves, the sensitivity and specificity for each outcome under study was plotted.

### Statistical analysis

Analyses were performed using SPSS 16.0 (Chicago, IL, USA). Categorical data were compared with Pearson chi squared tests or Fisher’s exact probability tests. Recurrence-free survival (RFS) was calculated as the time between the first day of diagnosis and the occurrence of local recurrence or distant metastasis. Overall survival (OS) was calculated as the time between the first day of diagnosis and the disease-related death. Survival probabilities were estimated by the Kaplan-Meier method and assessed by a log-rank test. The multivariate Cox proportional hazards regression model was used for assessing the association between potential confounding variables and prognosis (OS or RFS). Probability values ≤ 0.05 were considered statistically significant.

## Results

### Ano1 overexpression in breast cancer

**Fig 1:**
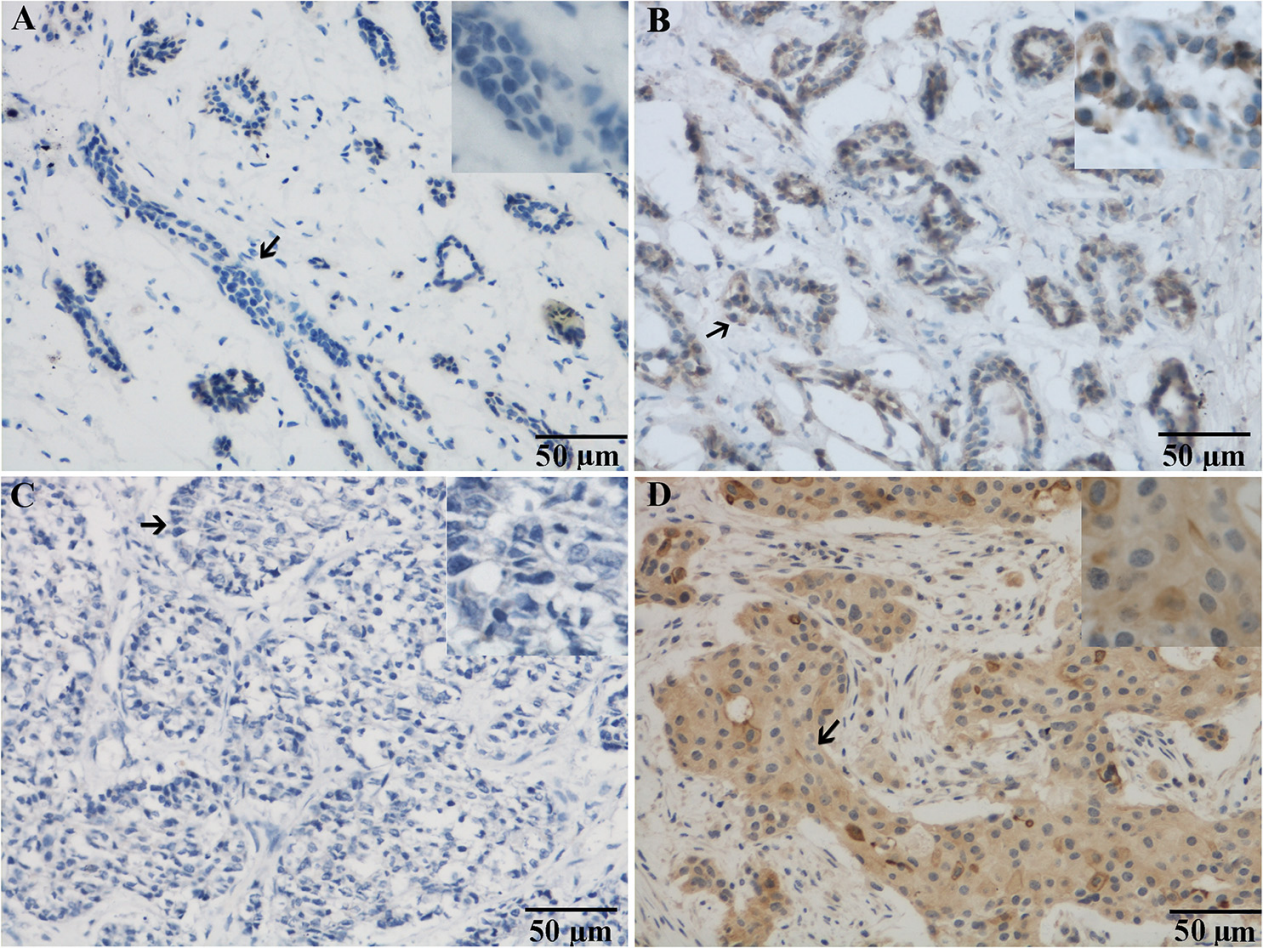
Representative micrographs showing negative (A,C) and positive (B,D) immunohistochemical staining of Ano1 in fibroadenoma (A, B) and breast cancer (C, D). Arrows indicate the magnified regions in the insert. Scale bar: 50 μm.

We studied the expression of Ano1 on 431 samples from patients with breast cancer and 46 samples from patients with fibroadenoma, using immunohistochemistry (Fig 1). Ano1 immunoreactivity was observed in 395 (91.6%) of 431 breast cancer samples. In contrast, Ano1 was found in 6 (13.0%) of 46 fibroadenoma samples. Ano1 immunoreactivity occurred significantly more frequently in breast cancer samples than in fibroadenoma samples (p<0.001).

### Clinicopathological characteristics of breast cancer patients with Ano1 expression

Table 1 summarizes clinicopathological characteristics of 395 breast cancer patients with Ano1 expression. The median age of these patients was 51 years (range, 20–82 years). The majority of these patients had a tumor with <5 cm in size, histological grade 2, and Stage I or II. Of the 395 patients, 268 (67.8%), 266 (67.3%), and 228 (57.5%) patients were ER-, PR-, and HER2-positive, respectively. Triple negative breast cancer (TNBC) patients were only recorded in 31 (7.8%) of 395 patients. 348 patients received anthracycline-based chemotherapy alone or in combination with paclitaxel. 305 patients were ER- and/or PR-positive, and all of them received tamoxifen treatment.

**Table 1:** Clinicopathological characteristics in breast cancer patients with Ano1 expression.

	No. of Cases	%
**Total no.**	395	100
**Median age [range], years**	51[20–82]	
**Age (years)**		
<51	210	53.2
≥51	185	46.8
**Sex**		
Female	395	100.0
**Menopausal status**		
Premenopausal	205	51.9
Postmenopausal	190	48.1
**First-degree family history of breast cancer**		
No	332	84.1
Yes	63	15.9
**Tumor size (cm)**		
≤ 2.0	134	33.9
<2 ~<5	201	50.9
≥5.0	60	15.2
**Histological grade**		
Grade 1	45	11.4
Grade 2	314	79.5
Grade 3	36	9.1
**Clinical stages**		
I or II	281	71.1
IIIA~IIIC	114	28.9
**Lymph node metastasis**		
Node-negative	194	49.1
Node-positive	201	50.9
**ER status**		
Negative	127	32.2
Positive	268	67.8
**PR status**		
Negative	129	32.7
Positive	266	67.3
**HER2 status**		
Negative	167	42.3
Positive	228	57.7
**Triple negative status**		
Non-triple negative	364	92.2
Triple negative	31	7.8
**Tamoxifen treatment**		
No	90	22.8
Yes	305	77.2
**Postoperative therapeutic regimens**		
Anthracycline alone or combined with paclitaxel	348	88.1
Other chemotherapies or treatments ^†^	47	11.9

Abbreviations: ER, Estrogen receptor; PR, Progesterone rec66eptor; HER2, Human epidermal growth factor receptor.

^†^Other chemotherapies included paclitaxel alone (*n* = 21), NP regimen (navelbine plus cisplatin, *n* = 15), TP regimen (Docetaxel plus cisplatin, *n* = 11).

### Selection of the cutoff value for Ano1expression

**Fig 2:**
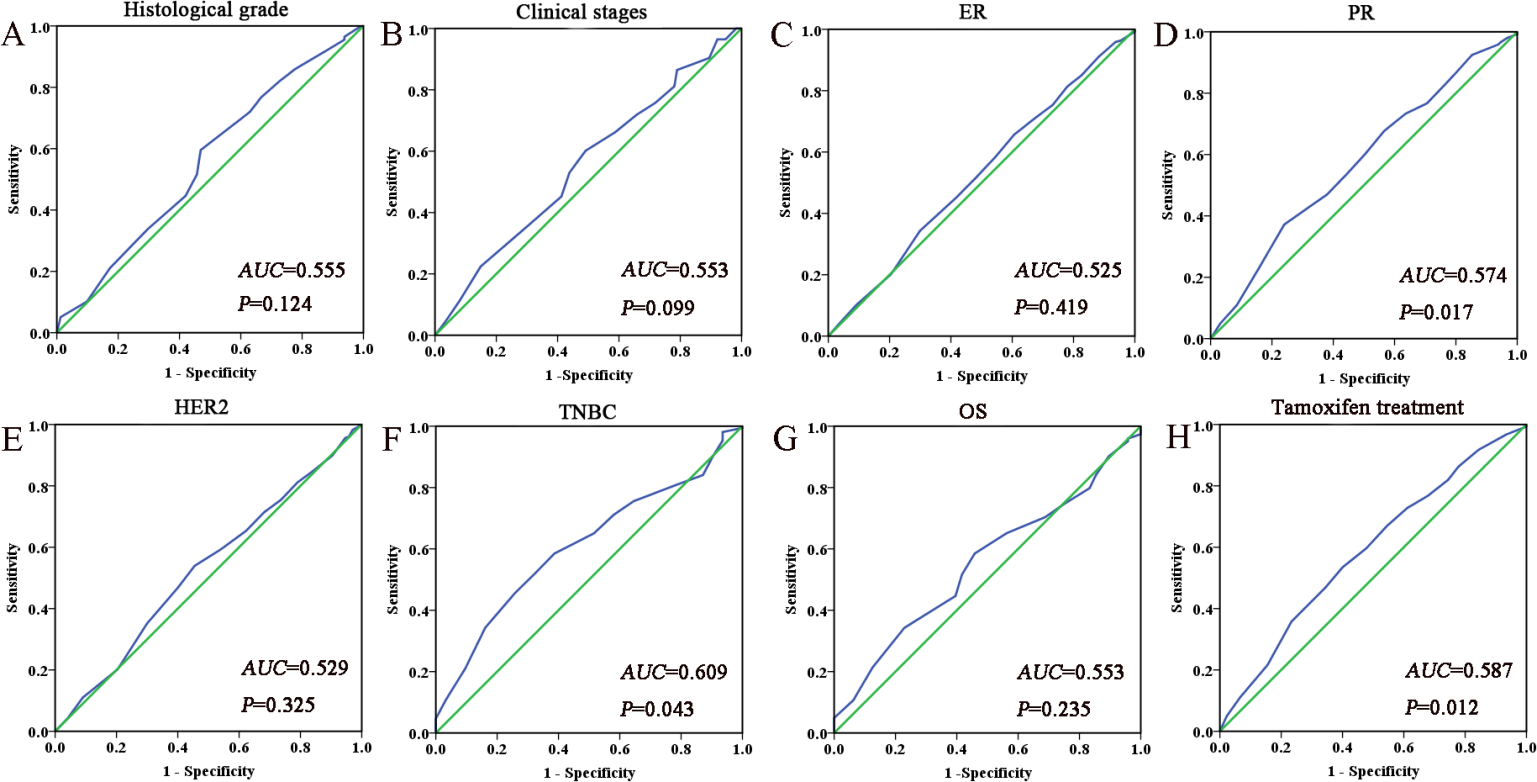
Receiver operating characteristic curves were used to determine the cutoff score for Ano1 overexpression in breast cancer. The sensitivity and specificity for each outcome were plotted and the areas under curve (AUCs) and p value were indicated. A. Histological grade; B. Clinical stages; C. ER status; D. PR status; E. HER2 status; F. TNBC status; G. OS; H. Tamoxifen treatment.

We performed the ROC analysis to determine an optimal cutoff score for Ano1expression. Fig 2 shows the ROC curves for each clinicopathological feature. ROC curves showed that the expression level of Ano1 was discriminated by the PR (p = 0.017), TNBC (p = 0.043) and tamoxifen treatment (p = 0.012). The AUC for the TNBC status had the biggest area (Fig 2F). Based on this outcome, a cutoff score of 65% were selected for Ano1 expression. Tumors with IHC scores >65% and ≤ 65% were defined as tumors with ‘high” and “low” expression of Ano1, respectively. 170 (43%) tumors exhibited the low expression of Ano1, and 225 (57%) tumors showed the high expression of Ano1.

### Association of Ano1 expression with clinicopathological characteristics of breast cancer

We then investigated the association between the Ano1 expression and clinicopathological characteristics of breast cancer (Table 2). The expression levels of Ano1 were not significantly associated with the patient’s age, menopausal status, family history, tumor size, histological grade, and lymph node metastasis (Table 2, p>0.05). Tumors with Stage I or II were more associated with the high expression of Ano1 (p = 0.045, Table 2). Furthermore, the high expression of Ano1 was more associated with grade 1 and 2 tumors with the ER-positive status(S1 Table), or stage I or II tumors with the HER2-negative status (S2 Table), but not in PR-positive (S3 Table) or PR-negative patients (S4 Table), and HER2 positive patients (S5 Table).

**Table 2:** Correlation of Ano1 expression with clinicopathological parameters in patients with breast cancer.

Characteristics	Low expression n (%)		High exression n (%)		*p* ^†^ ^,^ ^‡^	Adjusted OR (95%CI)^§^
**Age (years)**						
<51	87 (41.4)		123 (58.6)		0.491^†^	1 (reference)
≥51	83 (44.9)		102 (55.1)		0.879^‡^	1.069 (0.455–2.510)
**Menopausal status**						
Premenopausal	84 (41.0)		121 (59.0)		0.390^†^	1 (reference)
Postmenopausal	86 (45.3)		104 (54.7)		0.596^‡^	0.794 (0.339–1.863)
**First-degree family history of breast cancer**						
No	140 (42.2)		192 (57.8)		0.423^†^	1 (reference)
Yes	30 (47.6)		33 (52.4)		0.435^‡^	0.806 (0.469–1.385)
**Tumor size (cm)**						
≤ 2.0	55 (41.0)		79 (59.0)		0.762^†^	1 (reference)
<2 ~<5	87 (43.3)		114 (56.7)		0.520^‡^	0.817(0.441–1.514)
≥5.0	28 (46.7)		32 (53.3)		0.675^‡^	0.883 (0.494–1.579)
**Histological grade**						
Grade 1	24 (53.3)		21 (46.7)		0.123^†^	1 (reference)
Grade 2	127 (40.4)		187 (59.6)		0.945^‡^	1.032 (0.427–2.491)
Grade 3	19 (52.8)		17 (47.2)		0.166^‡^	0.612 (0.306–1.226)
**Clinical stage**						
I or II	112 (39.9)		169 (60.1)		**0.045** ^†^	1 (reference)
IIIA~IIIC	58 (50.9)		56 (49.1)		**0.045** ^‡^	**0.638 (0.411–0.990)**
**Lymph node metastasis**						
Node-negative	77 (39.7)		117 (60.3)		0.187^†^	1 (reference)
Node-positive	93 (46.3)		108 (53.7)		0.181^‡^	0.761 (0.510–1.136)
**ER status**						
Negative	58 (45.7)		69 (54.3)		0.467^†^	1 (reference)
Positive	112 (41.8)		156 (58.2)		0.490^‡^	1.162 (0.758–1.782)
**PR status**						
Negative	64 (49.6)		65 (50.4)		**0.066** ^†^	1 (reference)
Positive	106 (39.8)		160 (60.2)		**0.073** ^‡^	**1.483 (0.964–2.283)**
**HER2 status**						
Negative	77 (46.1)		90 (53.9)		0.292^†^	1 (reference)
Positive	93 (40.8)		135 (59.2)		0.243^‡^	1.274 (0.848–1.912)
**Triple negative status**						
Non-triple negative	151 (41.5)		213 (58.5)		**0.033** ^†^	1 (reference)
Triple negative	19 (61.3)		12 (38.7)		**0.031** ^‡^	**0.435 (0.204–0.925)**

Abbreviations: ER, Estrogen receptor; PR, Progesterone receptor; HER2, Human epidermal growth factor receptor.

^†^ *p* values were calculated from 2-sided chi-square tests or Fisher’s Exact Test.

^‡^ *p* values were calculated by unconditional logistic regression adjusted for age, menopause state.

^§^ OR and 95% CI values were calculated by unconditional logistic regression adjusted for age, menopause status, first degree family history of breast cancer.

The expression of Ano1 was significantly decreased in patients with triple-negative breast cancer (TNBC) (p = 0.033). PR-positive tumors exhibited a tendency toward higher Ano1 expression (p = 0.066). Ano1 expression was not significantly associated with the ER (Table 2). Although Ano1 expression was not significantly different between HER2-positive and HER2-negative breast cancer, Ano1 expression was significantly higher in breast cancer with HER2 IHC staining of 3+ compared with breast cancer with HER2 IHC staining of 0–1+ (S6 Table). Furthermore, we examined Ano1 expression in breast cancer patients with different ER, PR, and HER2 status (Table 3). In ER-negative patients, Ano1 expression was significantly higher in PR-positive tumors than in PR-negative tumors (p = 0.022, Table 3).

**Table 3:** Correlation of Ano1 expression with the ER, PR, and HER2 status.

Characteristics	Low expression n (%)	High expression n (%)	*p* ^†^ ^,^ ^‡^	Adjusted OR (95%CI)^§^
**ER-positive patients**				
PR status				
Negative	17 (43.6)	22 (56.4)	0.805†	1 (reference)
Positive	95 (41.5)	134 (58.5)	0.662‡	1.177 (0.567–2.443)
HER2 status				
Negative	51 (44.7)	63 (55.3)	0.400†	1 (reference)
Positive	61 (39.6)	93 (60.4)	0.377‡	1.249 (0.763–2.044)
**ER-negative patients**				
PR status				
Negative	47 (52.2)	43 (47.8)	**0.021** †	1 (reference)
Positive	11 (29.7)	26 (70.3)	**0.022** ‡	**2.694 (1.150–6.312)**
HER2 status				
Negative	26 (49.1)	27 (50.9)	0.517†	1 (reference)
Positive	32 (43.2)	42 (56.8)	0.408‡	1.365 (0.653–2.851)
**PR-positive patients**				
ER status				
Negative	11 (29.7)	26 (70.3)	0.175†	1 (reference)
Positive	95 (41.5)	134 (58.5)	0.133‡	0.556 (0.259–1.195)
HER2 status				
Negative	52 (42.6)	70 (57.4)	0.395†	1 (reference)
Positive	54 (37.5)	90 (62.5)	0.412‡	1.231 (0.750–2.021)
**PR-negative patients**				
ER status				
Negative	47 (52.2)	43 (47.8)	0.368†	1 (reference)
Positive	17 (43.6)	22 (56.4)	0.194‡	1.742 (0.754–4.026)
HER2 status				
Negative	25 (55.6)	20 (44.4)	0.323†	1 (reference)
Positive	39 (46.4)	45 (53.6)	0.251‡	1.546 (0.735–3.255)
**HER2-postive patients**				
ER status				
Negative	32 (43.2)	42 (56.8)	0.601†	1 (reference)
Positive	61 (39.6)	93 (60.4)	0.618‡	1.154 (0.656–2.031)
PR status				
Negative	39 (46.4)	45 (53.6)	0.186†	1 (reference)
Positive	54 (37.5)	90 (62.5)	0.169‡	1.475 (0.847–2.568)
**HER2-negative patients**				
ER status				
Negative	26 (49.1)	27 (50.9)	0.602†	1 (reference)
Positive	51 (44.7)	63 (55.3)	0.495‡	1.262 (0.647–2.461)
PR status				
Negative	25 (55.6)	20 (44.4)	0.137†	1 (reference)
Positive	52 (42.6)	70 (57.4)	0.155‡	1.671 (0.824–3.391)

Abbreviations: ER, Estrogen receptor; PR, Progesterone rec66eptor; HER2, Human epidermal growth factor receptor.

† *p* values were calculated from 2-sided chi-square tests or Fisher’s Exact Test.

‡ *p* values were calculated by unconditional logistic regression adjusted for age, menopause state.

§ OR and 95% CI values were calculated by unconditional logistic regression adjusted for age, menopause status, first degree family history of breast cancer.

### Association of the expression of Ano1 with the survival of breast cancer patients

**Fig 3:**
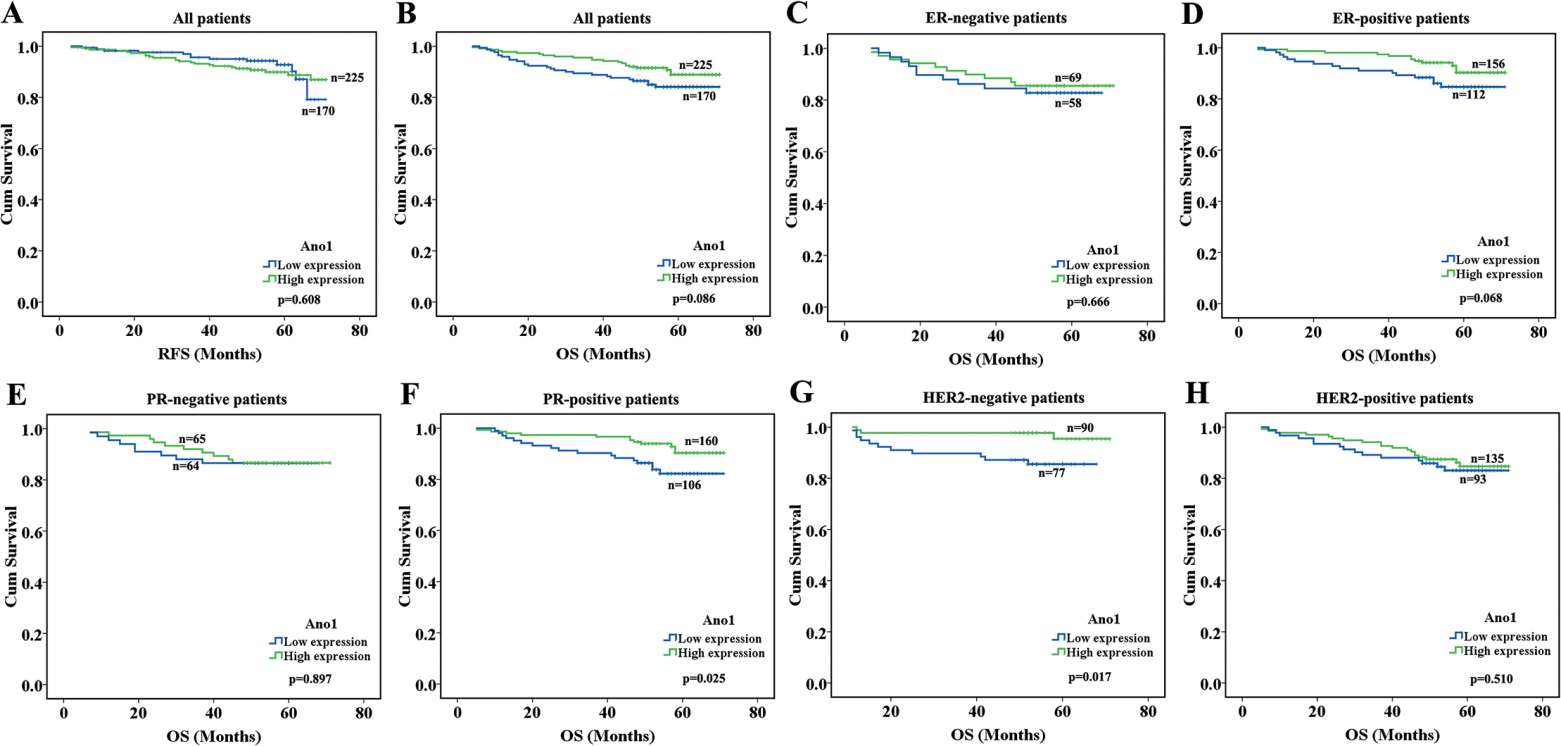
Kaplan-Meier survival analysis of Ano1 expression in breast cancer patients with different ER, PR, and HER2 status. The log-rank test was performed to test the statistical significance. A, B. Survival curves show the association between Ano1 expression and recurrence-free survival (RFS) (A) or overall survival (OS) (B) in 395 breast cancer patients with Ano1 expression. C-H. Survival curves show the association between Ano1 expression and OS in ER-negative patients (C), ER-positive patients (D), PR-negative patients (E), PR-positive patients (F), HER2-negative patients (G), and HER2-positive patients (H).

We evaluated the association of Ano1 expression levels with the OS and DFS in breast cancer patients, using Kaplan-Meier analysis. The high expression of Ano1 was associated with a tendency toward longer OS in breast cancer patients (n = 395, p = 0.086, Fig 3A and 3B). We further investigated the association of Ano1 expression with the OS and DFS in subgroups of breast cancer patients, categorized according to the ER, PR, and HER2 status. The high expression of Ano1 was associated with longer OS in patients with PR-positive (p = 0.025) or HER2-negative (p = 0.017) breast cancer (Fig 3C–3H).

**Fig 4:**
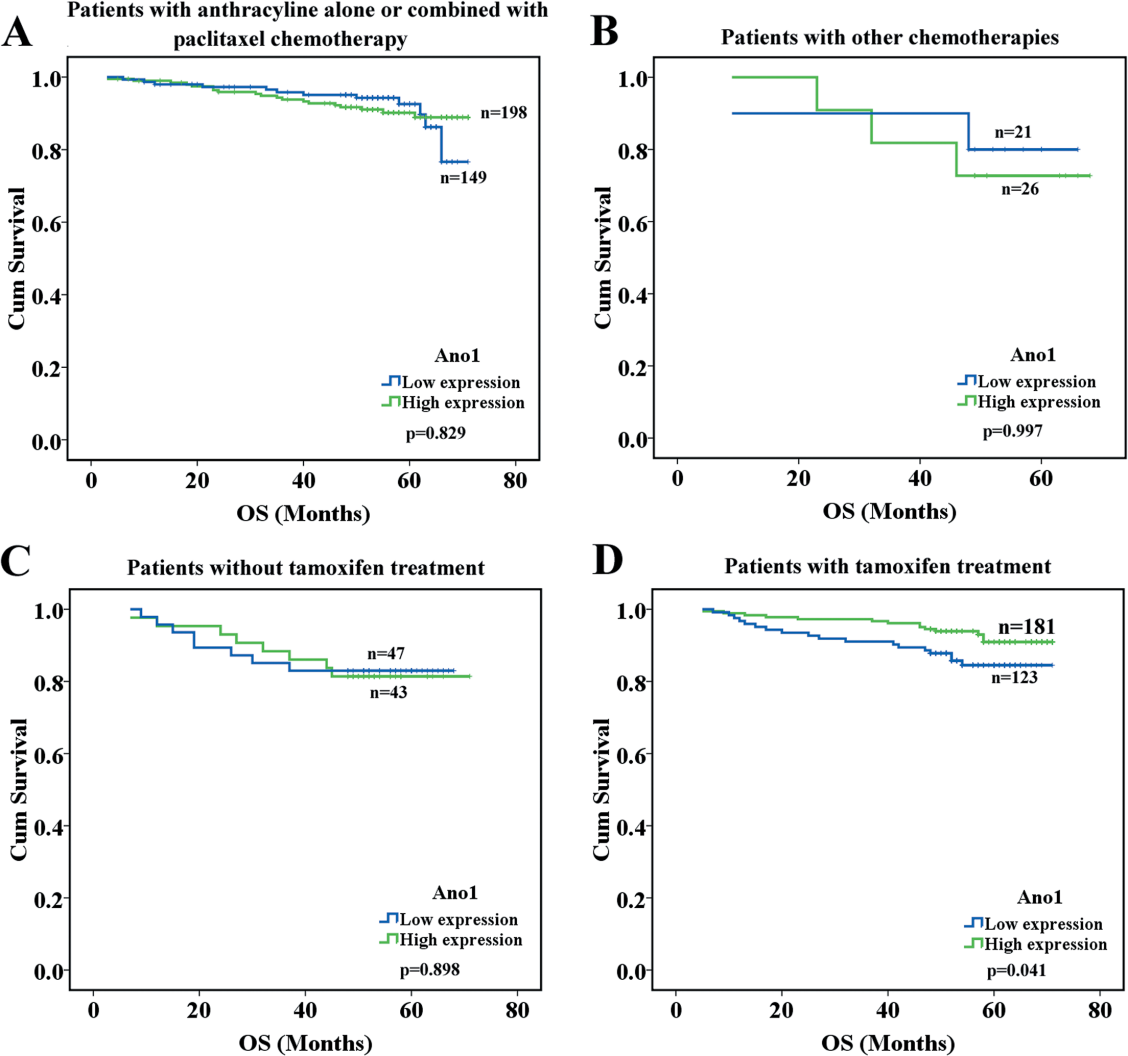
Kaplan-Meier survival analysis of Ano1 expression in breast cancer patients with chemotherapy or tamoxifen treatment. A,B. Survival curves show the association between Ano1 expression and overall survival (OS) in breast cancer patients receiving anthracycline alone or combined with paclitaxel (A) and other chemotherapies (B). C, D. Survival curves show the association between Ano1 expression and OS in breast cancer patients without (C) or with (D) tamoxifen treatment.

**Fig 5:**
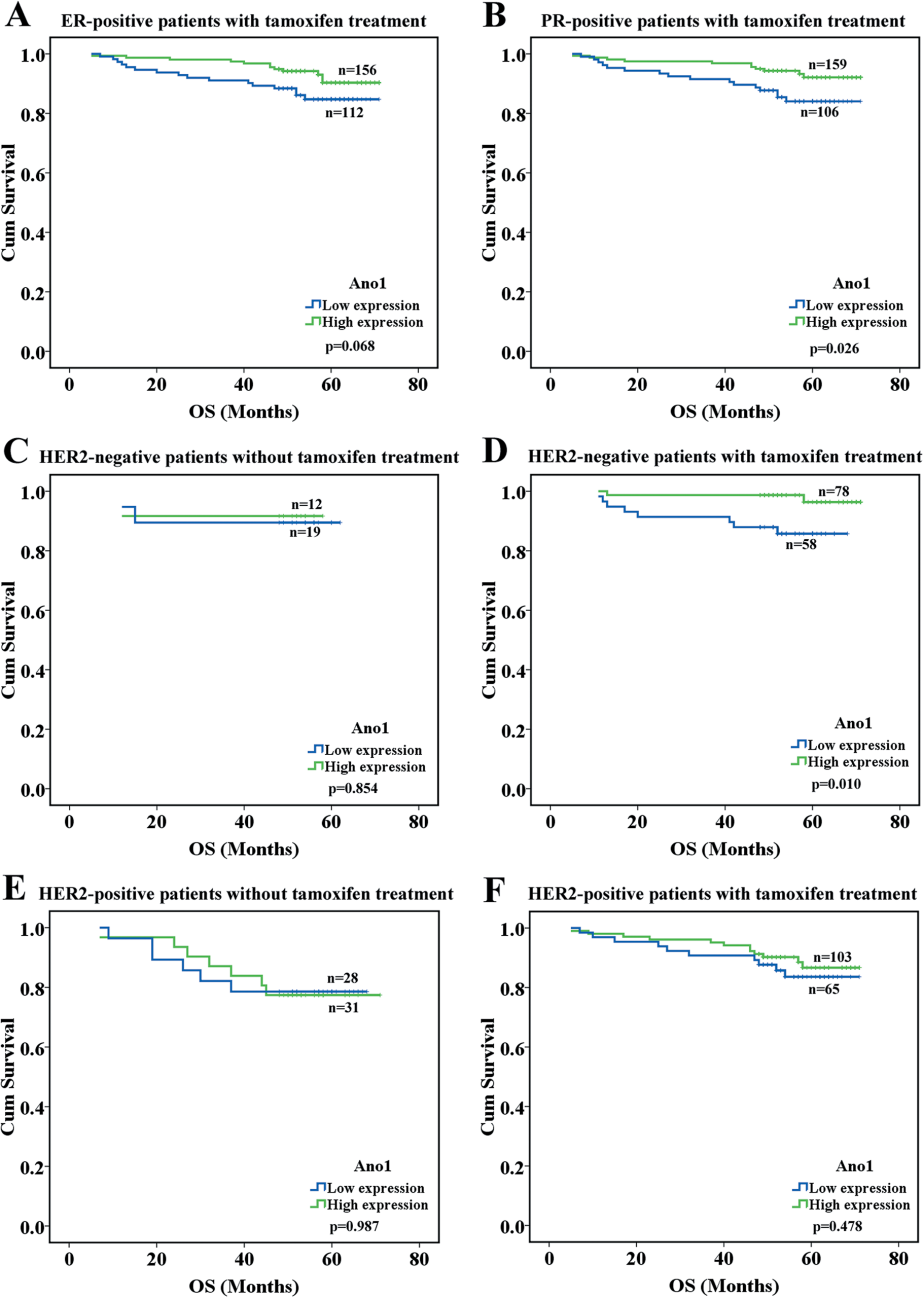
Kaplan-Meier survival analysis of Ano1 expression in breast cancer patients with different ER, PR, and HER2 status receiving tamoxifen treatment. Survival curves show the association between Ano1 expression and OS in ER-positive (A) and PR-positive (B) breast cancer patients with tamoxifen treatment, HER2-negative patients without (C) or with (D) tamoxifen treatment, and HER2-positive patients without (E) or with (F) tamoxifen treatment.

We then investigated the association between Ano1 expression levels and therapeutic responses in breast cancer patients receiving chemotherapy and endocrine therapy. Ano1 expression levels were not significantly associated with OS and RFS in breast cancer patients with chemotherapy (Fig 4A and 4B). The high expression of Ano1 was significantly associated with longer OS in patients receiving tamoxifen treatment (p = 0.041, Fig 4C and 4D). Although the high expression of Ano1 exhibited a tendency toward longer OS in ER-positive patients receiving tamoxifen treatment, no statistically significant difference was found (p = 0.068, Fig 5A). The high expression of Ano1 was significantly associated with longer OS in patients with PR-positive (p = 0.026), or HER2-negative (p = 0.010), but not HER2-postive (p = 0.478), breast cancer (Fig 5B–5F).

Multivariate Cox regression analysis was performed to evaluate the impact of Ano1 expression on the RFS and OS in breast cancer patients (Table 4). Ano1 overexpression was a prognostic factor for longer OS in PR-positive or HER2-negative patients, and a predictive factor for longer OS in patients following tamoxifen treatment. In addition, we also found that tumor size, clinical stage, and lymph node metastasis were prognostic factors for OS, and clinical stage was a prognostic factor for RFS in breast cancer patients.

**Table 4:** Multivariate COX regression analysis of the association of Ano1 expression and clinicopathological features with RFS and OS in breast cancer patients.

Survival	RFS				OS				
	Total n	Events n (%)	Adjusted HR (95%CI) ^†^	*p* ^†^	Total n	Events N (%)	Adjusted HR (95%CI) ^†^		*p* ^†^
**Ano1 Expression**									
ER positive patients									
Low	112	7 (6.3)	1 (reference)		112	16 (14.3)		1 (reference)	
High	156	18 (11.5)	1.758 (0.728–4.242)	0.209	156	12 (7.7)		0.497 (0.234–1.058)	0.070
ER negative patients									
Low	58	6 (10.3)	1 (reference)		58	10 (17.2)		1 (reference)	
High	69	5 (7.2)	0.575 (0.168–1.960)	0.376	69	10 (14.5)		0.869 (0.355–2.129)	0.759
PR positive patients									
Low	106	10 (9.4)	1 (reference)		106	16 (15.1)		1 (reference)	
High	160	16 (10.0)	0.966 (0.447–2.221)	0.993	160	11 (6.9)		**0.411 (0.190–0.893)**	**0.025**
PR negative patients									
Low	64	3 (4.7)	1 (reference)		64	10 (15.6)		1 (reference)	
High	65	7 (10.8)	1.986 (0.510–7.727)	0.322	65	11 (16.9)		1.018 (0.429–2.419)	0.967
HER2 positive patients									
Low	93	7 (7.5)	1 (reference)		93	16 (17.2)		1 (reference)	
High	135	14 (10.4)	1.292 (0.517–3.226)	0.583	135	19 (14.1)		0.800 (0.411–1.559)	0.512
HER2 negative patients									
Low	77	6 (7.8)	1 (reference)		77	10 (13.0)		1 (reference)	
High	90	9 (10.0)	1.100 (0.384–3.154)	0.859	90	3 (3.3)		**0.250 (0.068–0.920)**	**0.037**
Tamoxifen treatment									
Low	123	10 (8.1)	1 (reference)		123	18 (14.6)		1 (reference)	
High	182	20 (11.0)	1.268 (0.590–2.727)	0.543	182	14 (7.7)		**0.491 (0.243–0.990)**	**0.047**
Anthracycline alone or combined with Paclitaxel									
Low	149	12 (8.1)	1 (reference)		149	21 (14.1)		1 (reference)	
High	199	19 (9.5)	0.552 (0.137–2.227)	0.404	199	18 (9.0)		1.416 (0.373–5.373)	0.610
**Clininopathological features**									
Age (years)									
<51	210	23 (11.0)	1 (reference)		210	21 (10.0)		1 (reference)	
≥51	185	13 (7.0)	0.696 (0.191–2.535)	0.583	185	27 (14.6)		1.113 (0.350–3.539)	0.855
Menopausal status									
Premenopausal	205	22 (10.7)	1 (reference)		205	20 (9.8)		1 (reference)	
Postmenopausal	190	14 (7.4)	0.913 (0.255–3.266)	0.889	190	28 (14.7)		1.414 (0.442–4.529)	0.559
First-degree family history of cancer									
No	332	27 (8.1)	1 (reference)		332	39 (11.7)		1 (reference)	
Yes	63	9 (14.3)	1.609(0.753–3.434)	0.219	63	9 (14.3)		1.101 (0.532–2.279)	0.795
Tumor size (cm)									
≤ 2.0	134	11 (8.2)	1 (reference)		134	6 (4.5)		1 (reference)	
>2.0	261	25 (9.6)	1.109 (0.541–2.272)	0.778	261	42 (16.1)		**3.798 (1.611–8.953)**	**0.002**
Clinical stage									
I or II	281	21 (7.5)	1 (reference)		281	13 (4.6)		1 (reference)	
III or IV	114	15 (13.2)	**1.951 (1.002–3.797)**	**0.049**	114	35 (30.7)		**7.961 (4.203–15.076)**	**<0.001**
Lymph node metastasis									
Node-negative	194	14 (7.2)	1 (reference)		194	10 (5.2)		1 (reference)	
Node-positive	201	22 (10.9)	1.712 (0.874–3.353)	0.117	201	38 (18.9)		**4.030 (2.006–8.096)**	**<0.001**

Abbreviations: HR: Hazard Ratio; 95% CI, 95% confidence interval; RFS: Recurrence-free survival; OS: Overall survival; Ref, reference category; ER, Estrogen receptor; PR, Progesterone receptor; HER2, Human epidermal growth factor receptor.

^†^ *p* values, Adjusted HR (95%CI) were assessed using multivariate Cox regression analysis adjusted for age and menopause status.

## Discussion

Ano1 is overexpressed in many tumors, including breast cancer [15,22]. Consistent with these studies, we found that Ano1 was overexpressed in breast cancer compared with fibroadenoma. Ano1 was expressed in 91.6% of 432 breast cancer samples in our study, and in 78% of 49 breast cancer samples in a previous study by Britschgi et al. [15]. In contrast, we only detected Ano1 expression in 13.0% of 46 fibroadenoma samples. It has been reported that Ano1 is expressed in 2 (28.6%) of 7 cancer-adjacent normal breast tissues [13]. The findings that Ano1 is overexpressed in breast cancer compared with benign fibroadenoma and tumor-adjacent normal breast tissues suggest that Ano1 overexpression may contribute to malignant transformation of mammary epithelial cells into breast cancer. However, the reason for Ano1 overexpression in tumors remains unclear. Ano1 overexpression was found in many cancers with 11q13 amplification, including breast cancer [15]. However, Ano1 overexpression has also been found in many tumors without application of the 11q13 locus [25]. In addition, the 11q13 amplification only occurs in approximately 15%-20% of human breast cancer [31]. Therefore, the 11q13 amplification is not the only means to cause Ano1 overexpression, and signaling pathways or transcription factors may contribute to Ano1 overexpression during the tumorigenesis. ER and PR are the nuclear steroid receptors that regulate the transcriptional expression of many genes during breast cancer development [32]. In the present study, we identified that the expression of Ano1 was higher in PR-positive tumors compared with PR-negative tumors, especially in the ER-negative patients, suggesting that the ER and PR signaling pathways may be involved in Ano1 overexpression in breast cancer.

Although Ano1 is overexpressed in breast cancer, it remains unclear whether Ano1 overexpression promotes tumor development in breast cancer patients. In the present study, we found that Ano1 expression levels were not correlated with tumor size, histological grade, and lymph node metastasis in all breast cancer patients as well as those with different ER, PR, and HER2 status. Unexpectedly, we found that the high expression of Ano1 was more associated with less aggressive (stage I or II) HER2-negative tumors, or less differentiating (grade 1 or 2) ER-positive tumors (S1–S5 Tables). These results suggests that the high expression level of Ano1 predicates less aggressive behavior of ER-positive or HER-negative breast cancer. Since Ano1 overexpression did not correlate with clinical stage and histological grade in ER-negative, PR-positive, PR-negative, and HER2-postive tumors, cell-type specific mechanisms may be responsible for different roles of Ano1 in breast cancer cells with different ER, PR, and HER2 status. Cell-specific mechanisms may explain conflicting results in the literature regarding the role of Ano1 overexpression in cell proliferation and migration. For example, several *in vitro* studies have shown that Ano1 overexpression promotes cell proliferation and migration in head and neck squamous cell carcinoma, breast cancer, and prostate cancer [12,13,15,24]. However, Ubby et al. reported that breast cancer expressed various Ano1 isoforms, and overexpression of these Ano1 isoforms in HEK-293 cells did not promote cell proliferation and migration [22]. Furthermore, Ano1 inhibits angiotensin II-induced proliferation in basilar smooth muscle cells [26]. Since Ano1 is involved in a complex regulatory networks including the signaling/scaffolding proteins ezrin, radixin, and moesin, GTPases, Ca^2+^-binding proteins, kinases, and lipid-interacting proteins [33], it is not unexpected that Ano1 plays different roles in in different types of cells.

Tamoxifen is a “selective estrogen receptor modulator” that is widely used in endocrine therapy for breast cancer [34]. However, we found that although Ano1 overexpression exhibited a tendency toward a longer OS in ER-positive patients who received tamoxifen, no significant association was found between Ano1 overexpression and OS (p = 0.068, Fig 5A), suggesting that the primary effect of tamoxifen on Ano1 may not due to its inhibition on ER. Interestingly, tamoxifen has been found to inhibit chloride channels [35,36], including Ano1 [6], and inhibition of Ano1 currents reduces breast cancer cell survival [15]. In the present study, we found that Ano1 was higher in PR-positive breast cancer than in PR-negative breast cancer, and Ano1 overexpression was associated with good prognosis of PR-positive breast cancer patients following tamoxifen treatment. It is possible that inhibition of Ano1 channels by tamoxifen may contribute to the beneficial effect of tamoxifen treatment in PR-positive breast cancer patients. Furthermore, it has been reported that ER-negative but PR-positive patients gain substantial benefit from tamoxifen therapy [37], further suggesting that tamoxifen may exert beneficial effects via the PR signaling pathway independently on the ER. Our study suggests that Ano1 overexpression may be responsible for the beneficial effect of Ano1 in PR-positive patients, and may be a biomarker for good prognosis of PR-positive patients receiving tamoxifen.

In the present study, we found that overexpression of Ano1 proteins was associated with good prognosis in breast cancer patients following tamoxifen treatment, especially those with PR-positive or HER2-negative status. Since both Ano1 and ER can be inhibited by tamoxifen, the finding that Ano1 that has tumor-promoting properties is associated with good prognosis in breast cancer patients receiving tamoxifen resembles the fact that ER that promotes breast cancer growth is associated with good prognosis in patients following tamoxifen treatment [38]. However, the expression level of the Ano1 gene has been found to be associated with poor prognosis in breast cancer patients [15]. These seemly controversial results between the two studies may be due to different tamoxifen treatment conditions. Britschgi et al. found that patients with the low expression of the Ano1 gene mainly exhibited longer OS at the survival time >5 years [15], when breast cancer patients usually stop tamoxifen treatment [39]. It is possible that withdrawal of tamoxifen treatment may promote breast cancer development in patients with the genetic background of the high expression of Ano1 in the study by Britschgi et al. [15]. Tumors with the high expression of Ano1 are more likely to be inhibited by tamoxifen, and thus may lead to good prognosis of the patients with the high expression of Ano1 in our study.

A prognostic factor refers to a measurable variable that is associated with clinical outcomes in the absence of therapy, and correlated with the nature history of the disease. Thus, the finding that Ano1 overexpression was associated with poor prognosis in breast cancer patients with 11q13 amplification in the study by Britschgi et al. [15] suggests that Ano1 overexpression is a prognostic factor for this specific population of breast cancer. In contrast, a predictive factor is the one that is associated with clinical responses to a given therapy. Our finding that Ano1 overexpression was associated with good prognosis in PR-positive or HER2-negative breast cancer patients following tamoxifen treatment suggests that Ano1 overexpression is a predictive factor for tamoxifen benefit in these subgroups of breast cancer patients. Therefore, Ano1 expression levels have both prognostic and predictive values for breast cancer patients.

We also found that Ano1 expression did not correlate with the HER2 status (Table 2), suggesting that HER2 signaling was not likely to contribute to Ano1 overexpression. In addition, we found that Ano1 overexpression was associated with longer OS in HER2-negative, but not HER2-postive, patients following tamoxifen treatment, suggesting that HER2 signaling may be involved in the role of Ano1 in breast cancer development. Ano1 has been found to activate the EGFR signaling pathway, and promotes breast cancer tumorigenesis [15]. EGFR is commonly associated with overexpression of HER2, a member of the EGFR family [40]. In HER2-postive breast cancer, it is possible that excessive activation of HER2 predominately controls the signaling pathway, thus masking the activation of EGFR signaling by Ano1. In HER2-negative breast cancer, EGFR signaling activation by Ano1 may contribute to breast cancer tumorigenesis, and thus Ano1 inhibition by tamoxifen may result in good prognosis in HER2-negative patients receiving tamoxifen treatment. Furthermore, the multivariate Cox analysis showed that Ano1 overexpression was significantly associated with longer OS in HER2-negative patients, further suggesting that Ano1 has a prognostic value in HER2-negative patients.

Although the best method to assess HER2 status remains controversial, it is generally accepted that breast cancer patients with HER2 overexpression by IHC (3+) and gene amplification ratio of >2 by fluorescent in situ hybridization (FISH) were considered for adjuvant trastuzumab treatment [41]. Currently, the treatment rate with trastuzumab for HER2-positive breast cancer in China is low (approximately 30%) due to high treatment costs and insufficient insurance coverage [42]. To exclude the effect of trastuzumab on the survival of breast cancer patients, we excluded a few patients who received adjuvant trastuzumab treatment (n = 35). Since this study did not include patients with trastuzumab treatment and we aimed to study the association between Ano1 expression and HER2 expression, we defined HER2-postive status by 2+ or 3+ staining, instead of 3+ staining for anti-HER2 therapy. This definition led to a high HER2-postive rate (57.7%) in the present study, which is higher than the rate (approximately 15–20%) reported in the literature determined by IHC staining of 3+ [43].

Although our study is strengthened by a relatively large sample size, it also has some limitations. Although we found that the expression of Ano1 in PR-positive tumors was higher than in PR-negative tumors, especially in the ER-negative tumors, it remains to be determined whether Ano1 expression is regulated by the ER/PR signaling pathways. Recently, Matsuba et al. reported that inhibition of histone deacetylase (HDAC) downregulated the expression of Ano1 in breast cancer cells [44]. It has been reported that inhibition of HDAC silences PR-mediated signaling [45]. Further studies are required to identify whether PR signaling is involved in HDAC-mediated regulation of Ano1 expression. In addition, although the 11q13 amplification contributes to Ano1 overexpression in human breast cancer [15], it is unlikely the major contributor to Ano1 overexpression in PR-positive tumors, because the 11q13 amplification only occurs in approximately 15% of human breast cancer [34]. However, we can not exclude the possibility that 11q13 amplification plays a role in Ano1 overexpression in PR-positive tumors, since 11q13 has been identified as a susceptibility locus for ER- and PR-positive breast cancer [46]. Our finding can be strengthened by investigating the 11q13 status in the PR-positive cohort vs PR-negative cohort.

In summary, although many *in vitro* studies have demonstrated that Ano1 promotes tumorigenesis [12,13,15,24], the role of Ano1 in tumorigenesis remains unclear and conflicting results exist in the literature regarding the role of Ano1 in proliferation and migration of different cells. Regulation of tumorigenesis by Ano1 may be associated with a cell-type specific mechanism in which Ano1 expression and function may depend on additional factors. This cell-type specificity might determine different responses of cells to overexpression or knockdown of Ano1 [12,15,22,26], and thus may contribute to different therapeutic responses to inhibition or activation of Ano1. In the present study, we found that the high expression of Ano1 was associated with good prognosis in PR-positive or HER2-negative breast cancer patients following tamoxifen treatment. Our findings suggest the PR and HER2 status can define a subtype of breast cancer in which Ano1 overexpression is associated with good prognosis in patients receiving tamoxifen treatment.

## Supporting Information

S1 Table Correlation of Ano1 expression with clinicopathological parameters in ER-positive patients. (DOCX)

S2 Table Correlation of Ano1 expression with clinicopathological parameters in ER-negative patients. (DOCX)

S3 Table Correlation of Ano1 expression with clinicopathological parameters in PR-positive patients. (DOCX)

S4 Table Correlation of Ano1 expression with clinicopathological parameters in PR-negative patients. (DOCX)

S5 Table Correlation of Ano1 expression with clinicopathological parameters in HER2 positive patients. (DOCX)

S6 Table The expression of Ano1 in breast cancer patients with different HER2 IHC scores. (DOCX)
