# Supplementary material for: Ano1/TMEM16A Overexpression Is Associated with Good Prognosis in PR-Positive or HER2-Negative Breast Cancer Patients following Tamoxifen Treatment
Source: PLoS One. 2015 May 11;10(5):e0126128. doi: 10.1371/journal.pone.0126128 (PMC4427473; doi:10.1371/journal.pone.0126128)
Supplement: S1 Table — (DOCX) [file pone.0126128.s001.docx]

**S1 Table. Correlation of Ano1 expression with clinicopathological parameters in ER-positive patients.**

|  | **Ano1 expression** | | | |
| --- | --- | --- | --- | --- |
|  | **Low**  **n (%)** | **High**  **n (%)** | ***p* value**^†,‡^ | **OR (95%CI)^§^** |
| **Age, yrs** |  |  |  |  |
| <51 | 57 (41.0) | 82 (59.0) | 0.787^†^ | 1 (reference) |
| ≥51 | 55 (42.6) | 74 (57.4) | 0.609^‡^ | 0.763 (0.271-2.151) |
| **Menopausal status** |  |  |  |  |
| Premenopausal | 57 (41.6) | 80 (58.4) | 0.950^†^ | 1 (reference) |
| Postmenopausal | 55 (42.0) | 76 (58.0) | 0.696^‡^ | 1.229 (0.437-3.457) |
| **First-degree family history of breast cancer** | | | |  |
| No | 93 (40.4) | 137 (59.6) | 0.268^†^ | 1 (reference) |
| Yes | 19 (50.0) | 19 (50.0) | 0.258^‡^ | 0.671 (0.337-1.339) |
| **Tumor size (cm)** |  |  |  |  |
| ≤ 2.0 | 36 (38.7) | 57 (61.3) | 0.456^†^ | 1 (reference) |
| >2.0 | 76 (43.4) | 99 (56.6) | 0.511^‡^ | 0.841 (0.502-1.410) |
| **Histological grade** |  |  |  |  |
| Grade 1 | 17 (54.8) | 14 (45.2) | **0.029**^†^ | 1 (reference) |
| Grade 2 | 85 (38.3) | 137 (61.7) | 0.442^‡^ | 0.600 (0.163-2.205) |
| Grade 3 | 10 (66.7) | 5 (33.3) | **0.036^‡^** | **0.300 (0.097-0.922)** |
| **Clinical stages** |  |  |  |  |
| I or II | 73 (38.4) | 117 (61.6) | 0.081^†^ | 1 (reference) |
| IIIA~IIIC | 39 (50.0) | 39 (50.0) | 0.083^‡^ | 0.624 (0.365-1.064) |
| **Lymph node metastasis** | |  |  |  |
| Node-negative | 48 (37.5) | 80 (62.5) | 0.173^†^ | 1 (reference) |
| Node-positive | 64 (45.7) | 76 (54.3) | 0.170^‡^ | 0.710 (0.435-1.159) |

^†^ *p* values were calculated from 2-sided chi-square tests or Fisher’s exact test.

^‡^*p* values were calculated by unconditional logistic regression adjusted for age, menopause state.

^§^ OR and 95% CI values were calculated by unconditional logistic regression adjusted for age, menopause status, first degree family history of breast cancer.
