# Supplementary material for: Ano1/TMEM16A Overexpression Is Associated with Good Prognosis in PR-Positive or HER2-Negative Breast Cancer Patients following Tamoxifen Treatment
Source: PLoS One. 2015 May 11;10(5):e0126128. doi: 10.1371/journal.pone.0126128 (PMC4427473; doi:10.1371/journal.pone.0126128)
Supplement: S2 Table — (DOCX) [file pone.0126128.s002.docx]

**S2 Table. Correlation of Ano1 expression with clinicopathological parameters in ER-negative patients.**

|  | **Ano1 expression** | | | |
| --- | --- | --- | --- | --- |
|  | **Low**  **n (%)** | **High**  **n (%)** | ***p* value**^†,‡^ | **OR (95%CI)^§^** |
| **Age, y** |  |  |  |  |
| <51 | 30 (42.3) | 41 (57.7) | 0.384^†^ | 1 (reference) |
| ≥51 | 28 (50.0) | 28 (50.0) | 0.375^‡^ | 2.162 (0.393-11.878) |
| **Menopausal status** |  |  |  |  |
| Premenopausal | 27 (39.7) | 41 (60.3) | 0.148^†^ | 1 (reference) |
| Postmenopausal | 31 (52.5) | 28 (47.5) | 0.159^‡^ | 0.297 (0.055-1.609) |
| **First-degree family history of breast cancer** | | |  |  |
| No | 47 (46.1) | 55 (53.9) | 0.852^†^ | 1 (reference) |
| Yes | 11 (44.0) | 14 (56.0) | 0.758^‡^ | 1.153 (0.466-2.848) |
| **Tumor size (cm)** |  |  |  |  |
| ≤ 2.0 | 19 (46.3) | 22 (53.7) | 0.916^†^ | 1 (reference) |
| >2.0 | 39 (45.3) | 47 (54.7) | 0.869^‡^ | 1.066 (0.499-2.277) |
| **Histological grade** |  |  |  |  |
| Grade 1 | 7 (50.0) | 7 (50.0) | 0.917^†^ | 1 (reference) |
| Grade 2 | 42 (45.7) | 50 (54.3) | 0.734^‡^ | 1.271 (0.318-5.091) |
| Grade 3 | 9 (42.9) | 12 (57.1) | 0.964^‡^ | 1.023 (0.382-2.739) |
| **Clinical stages** |  |  |  |  |
| I or II | 39 (42.9) | 52 (57.1) | 0.312^†^ | 1 (reference) |
| IIIA~IIIC | 19 (52.8) | 17 (47.2) | 0.217^‡^ | 0.606 (0.273-1.343) |
| **Lymph node metastasis** |  |  |  |  |
| Node-negative | 29 (43.9) | 37 (56.1) | 0.684^†^ | 1 (reference) |
| Node-positive | 29 (47.5) | 32 (52.5) | 0.683^‡^ | 0.862 (0.424-1.756) |

^†^ *p* values were calculated from 2-sided chi-square tests or Fisher’s exact test.

^‡^*p* values were calculated by unconditional logistic regression adjusted for age, menopause state.

^§^ OR and 95% CI values were calculated by unconditional logistic regression adjusted for age, menopause status, first degree family history of breast cancer.
