# Supplementary material for: Ano1/TMEM16A Overexpression Is Associated with Good Prognosis in PR-Positive or HER2-Negative Breast Cancer Patients following Tamoxifen Treatment
Source: PLoS One. 2015 May 11;10(5):e0126128. doi: 10.1371/journal.pone.0126128 (PMC4427473; doi:10.1371/journal.pone.0126128)
Supplement: S3 Table — (DOCX) [file pone.0126128.s003.docx]

**S3 Table. Correlation of Ano1 expression with clinicopathological parameters in PR-positive patients.**

|  | **Ano1 expression** | | | |
| --- | --- | --- | --- | --- |
|  | **Low**  **n (%)** | **High**  **n (%)** | ***p* value**^†,‡^ | **OR (95%CI)^§^** |
| **Age, y** |  |  |  |  |
| <51 | 64 (41.3) | 91 (58.7) | 0.571^†^ | 1 (reference) |
| ≥51 | 42 (37.8) | 69 (62.2) | 0.474^‡^ | 1.466 (0.515-4.177) |
| **Menopausal status** |  |  |  |  |
| Premenopausal | 62 (40.5) | 91 (59.5) | 0.794^†^ | 1 (reference) |
| Postmenopausal | 44 (38.9) | 69 (61.1) | 0.616^‡^ | 0.766 (0.271-2.171) |
| **First-degree family history of breast cancer** | | |  |  |
| No | 85 (38.8) | 134 (61.2) | 0.456^†^ | 1 (reference) |
| Yes | 21 (44.7) | 26 (55.3) | 0.449^‡^ | 0.782 (0.413-1.478) |
| **Tumor size (cm)** |  |  |  |  |
| ≤ 2.0 | 35 (40.2) | 52 (59.8) | 0.930^†^ | 1 (reference) |
| >2.0 | 71 (39.7) | 108 (60.3) | 0.849^‡^ | 1.053 (0.622-1.782) |
| **Histological grade** |  |  |  |  |
| Grade 1 | 16 (48.5) | 17 (51.5) | 0.072^†^ | 1 (reference) |
| Grade 2 | 81 (37.0) | 138 (63.0) | 0.343^‡^ | 0.535 (0.146-1.952) |
| Grade 3 | 9 (64.3) | 5 (35.7) | 0.053^‡^ | 0.326 (0.105-1.012) |
| **Clinical stages** |  |  |  |  |
| I or II | 69 (36.7) | 119 (63.3) | 0.104^†^ | 1 (reference) |
| IIIA~IIIC | 37 (47.4) | 41 (52.6) | 0.117^‡^ | 0.651 (0.381-1.114) |
| **Lymph node metastasis** |  |  |  |  |
| Node-negative | 47 (35.9) | 84 (64.1) | 0.192^†^ | 1 (reference) |
| Node-positive | 59 (43.7) | 76 (56.3) | 0.206^‡^ | 0.727 (0.443-1.192) |

^†^ *p* values were calculated from 2-sided chi-square tests or Fisher’s exact test.

^‡^*p* values were calculated by unconditional logistic regression adjusted for age, menopause state.

^§^ OR and 95% CI values were calculated by unconditional logistic regression adjusted for age, menopause status, first degree family history of breast cancer.
