# Supplementary material for: Ano1/TMEM16A Overexpression Is Associated with Good Prognosis in PR-Positive or HER2-Negative Breast Cancer Patients following Tamoxifen Treatment
Source: PLoS One. 2015 May 11;10(5):e0126128. doi: 10.1371/journal.pone.0126128 (PMC4427473; doi:10.1371/journal.pone.0126128)
Supplement: S4 Table — (DOCX) [file pone.0126128.s004.docx]

**S4 Table. Correlation of Ano1 expression with clinicopathological parameters in PR-negative patients.**

|  | **Ano1 expression** | | | |
| --- | --- | --- | --- | --- |
|  | **Low**  **n (%)** | **High**  **n (%)** | ***p* value**^†,‡^ | **OR (95%CI)^§^** |
| **Age, y** |  |  |  |  |
| <51 | 23 (41.8) | 32 (58.2) | 0.127^†^ | 1 (reference) |
| ≥51 | 41 (55.4) | 33 (44.6) | 0.475^‡^ | 0.568(0.121-2.677) |
| **Menopausal status** |  |  |  |  |
| Premenopausal | 22 (42.3) | 30(57.7) | 0.173^†^ | 1 (reference) |
| Postmenopausal | 42 (54.5) | 35(45.5) | 0.993^‡^ | 0.993(0.207-4.757) |
| **First-degree family history of breast cancer** | | |  |  |
| No | 55 (48.7) | 58(51.3) | 0.570^†^ | 1 (reference) |
| Yes | 9 (56.2) | 7(43.8) | 0.658^‡^ | 0.785(0.269-2.291) |
| **Tumor size (cm)** |  |  |  |  |
| ≤ 2.0 | 20 (42.6) | 27 (57.4) | 0.225^†^ | 1 (reference) |
| >2.0 | 44 (53.7) | 38 (46.3) | 0.313^‡^ | 0.685(0.328-1.429) |
| **Histological grade** |  |  |  |  |
| Grade 1 | 8 (66.7) | 4 (33.3) | 0.449^†^ | 1 (reference) |
| Grade 2 | 46 (48.4) | 49 (51.6) | 0.246^‡^ | 0.468(0.130-1.687) |
| Grade 3 | 10 (45.5) | 12 (54.5) | 0.274^‡^ | 0.463(0.099-1.929) |
| **Clinical stages** |  |  |  |  |
| I or II | 43 (46.2) | 50 (53.8) | 0.218^†^ | 1 (reference) |
| IIIA~IIIC | 21 (58.3) | 15 (41.7) | 0.188^‡^ | 0.588(0.267-1.296) |
| **Lymph node metastasis** |  |  |  |  |
| Node-negative | 30 (47.6) | 33 (52.4) | 0.658^†^ | 1 (reference) |
| Node-positive | 34 (51.5) | 32 (48.5) | 0.627^‡^ | 0.840(0.416-1.695) |

^†^ *p* values were calculated from 2-sided chi-square tests or Fisher’s exact test.

^‡^*p* values were calculated by unconditional logistic regression adjusted for age, menopause state.

^§^ OR and 95% CI values were calculated by unconditional logistic regression adjusted for age, menopause status, first degree family history of breast cancer.
