# Supplementary material for: Ano1/TMEM16A Overexpression Is Associated with Good Prognosis in PR-Positive or HER2-Negative Breast Cancer Patients following Tamoxifen Treatment
Source: PLoS One. 2015 May 11;10(5):e0126128. doi: 10.1371/journal.pone.0126128 (PMC4427473; doi:10.1371/journal.pone.0126128)
Supplement: S5 Table — (DOCX) [file pone.0126128.s005.docx]

**S5 Table. Correlation of Ano1 expression with clinicopathological parameters in HER2 positive patients.**

|  | **Ano1 expression** | | | |
| --- | --- | --- | --- | --- |
|  | **Low**  **n (%)** | **High**  **n (%)** | ***p* value**^†,‡^ | **OR (95%CI)^§^** |
| **Age, y** |  |  |  |  |
| <51 | 46 (41.1) | 66 (58.9) | 0.932^†^ | 1 (reference) |
| ≥51 | 47 (40.5) | 69 (59.5) | 0.898^‡^ | 1.081(0.329-3.556) |
| **Menopausal status** |  |  |  |  |
| Premenopausal | 45 (40.9) | 65 (59.1) | 0.972^†^ | 1 (reference) |
| Postmenopausal | 48 (40.7) | 70 (59.3) | 0.927^‡^ | 0.946(0.289-3.093) |
| **First-degree family history of breast cancer** | | |  |  |
| No | 76 (40.0) | 112 (59.6) | 0.808^†^ | 1 (reference) |
| Yes | 17 (42.5) | 23 (57.5) | 0.801^‡^ | 0.914(0.455-1.837) |
| **Tumor size (cm)** |  |  |  |  |
| ≤ 2.0 | 26 (36.1) | 46 (63.9) | 0.329^†^ | 1 (reference) |
| >2.0 | 67 (42.9) | 89 (57.1) | 0.335^‡^ | 0.752(0.422-1.342) |
| **Histological grade** |  |  |  |  |
| Grade 1 | 14 (56.0) | 11 (44.0) | 0.122^†^ | 1 (reference) |
| Grade 2 | 66 (37.3) | 111 (62.7) | 0.078^‡^ | 0.467(0.200-1.089) |
| Grade 3 | 13 (50.0) | 13 (50.0) | 0.670^‡^ | 0.787(0.261-2.375) |
| **Clinical stages** |  |  |  |  |
| I or II | 64 (39.0) | 100 (61.0) | 0.385^†^ | 1 (reference) |
| IIIA~IIIC | 29 (45.3) | 35 (54.7) | 0.394^‡^ | 0.775(0.431-1.393) |
| **Lymph node metastasis** |  |  |  |  |
| Node-negative | 44 (37.9) | 72 (62.1) | 0.371^†^ | 1 (reference) |
| Node-positive | 49 (43.8) | 63 (56.2) | 0.380^‡^ | 0.788(0.463-1.342) |

^†^ *p* values were calculated from 2-sided chi-square tests or Fisher’s exact test.

^‡^*p* values were calculated by unconditional logistic regression adjusted for age, menopause state.

^§^ OR and 95% CI values were calculated by unconditional logistic regression adjusted for age, menopause status, first degree family history of breast cancer.
