# Supplementary material for: Ano1/TMEM16A Overexpression Is Associated with Good Prognosis in PR-Positive or HER2-Negative Breast Cancer Patients following Tamoxifen Treatment
Source: PLoS One. 2015 May 11;10(5):e0126128. doi: 10.1371/journal.pone.0126128 (PMC4427473; doi:10.1371/journal.pone.0126128)
Supplement: S6 Table — (DOCX) [file pone.0126128.s006.docx]

**S6 Table. The expression of Ano1 in breast cancer patients with different HER2 IHC scores.**

| **IHC score** | **No.** | **%** | **Ano1 expression** | | ***p* value**^†,‡^ | **Adjusted**  **OR (95% CI)^§^** |
| --- | --- | --- | --- | --- | --- | --- |
|  |  |  | **Low**  ***n* (%)** | **High**  ***n* (%)** |  |  |
| 0-1+ | 167 | 42.3 | 77 (46.1) | 90 (53.9) | 0.028^†^ | 1 (reference) |
| 2+ | 117 | 29.6 | 56 (47.9) | 61 (52.1) | 0.918^‡^ | 1.025 (0.636-1.652) |
| 3+ | 111 | 28.1 | 36 (32.4) | 75 (67.6) | 0.013^‡^ | 0.528 (0.318-0.875) |

**Abbreviations**: HER2, Human epidermal growth factor receptor.

^†^*p* values were calculated from 2-sided chi-square tests or Fisher’s exact test.

^‡^*p* values were calculated by unconditional logistic regression adjusted for age, menopause state.

^§^OR and 95% CI values were calculated by unconditional logistic regression adjusted for age, menopause status, first degree family history of breast cancer.
